# Book Review

**Published:** 1898-03

**Authors:** 


**An Account of the Life and Works of Dr. Robert Watt.** By James Finlayson, M.D. Pp. 46. London: Smith, Elder, & Co. 1897.—The subject of this essay is probably better known to the public and possibly to the medical profession as the author of the *Bibliotheca Britannica*, than as one of the leading physicians of his time at Glasgow. Dr. Watt, like so many of his countrymen who have distinguished themselves in the world of literature and art, rose from a very humble position chiefly by his own efforts. Beginning life behind the plough, he raised himself to a position of no small reputation as a physician and lecturer on medicine, while from his pen came many useful and thoughtful books and papers on a variety of subjects connected with his profession. During the seventeen years that he practised he was constantly and with the greatest diligence employed in collecting materials for his great work, the publication of which he was deprived of seeing by his early death at the age of forty-four. Though the work was almost completely in MS., it appears to have been only by a most strange series of coincidences that the book was ever published. Dr. Finlayson has made a very readable little essay on Dr. Watt's life and work, and has adorned it with an admirable photogravure of his subject, after a portrait attributed to Sir Henry Raeburn.

**Problems of Nature: Researches and Discoveries of Gustav Jaeger, M.D.** Edited and translated by HENRY G. SCHLICHTER, D.Sc. Pp. x., 261. London: Williams and Norgate. 1897.—To many Englishmen it will be news that the author of these essays, whose name is connected so prominently with various articles of clothing, is a worker in many branches of organic science. The essays here collected range over an extensive area of investigation, and deal with the doctrine of evolution, and questions such as training and exercise, the treatment of infectious disease, and the configuration of the arctic regions. The papers were originally published a long time ago and then had no doubt considerable value, and now in their English dress may still be read with interest.

**Moods: Their Mental and Physical Character.** [F. PHILLIPS]. Pp. 16. London: J. & A. Churchill. 1897.—This little pamphlet is apparently written with the object of pointing out that many of our "moods" depend on physical conditions; also to tell medical men that "mental cases" are imperfectly understood. If we are to be reminded of such obvious truisms, it ought to be done by someone who can claim a knowledge of the subject and an intelligible style of expression. These do not seem to be possessed by the author of this confusing paper.

**Influenza.** By WILLIAM GRAY, M.D. Pp. 71. London: H. K. Lewis. 1897.—Dealing with influenza from a clinical point of view, Dr. Gray here gives us his experience, which has extended over a large number of cases most carefully observed. The parts most worthy of notice are the description of influenza in childbed, and its diagnosis from puerperal septicæmia: the author finds that both are liable to occur between two and three days after confinement, but in puerperal septicæmia the onset is insidious and the symptoms gradually increase in severity, whilst the lochia are generally suppressed or altered in character, the milk-secretion is often arrested, and there are symptoms more or less referable to a pelvic origin; in influenza the onset is sudden, the duration of the acute stage short and the crisis rapid, whilst the lochia as a rule are not suppressed and there is no foul odour, and the milk-secretion is not materially interfered with. There are also valuable remarks on the scarlatiniform rash which may occur; and on the average interval between an attack and subsequent relapse. The book is a good instance of the importance of carefully kept records in private practice, and of the value of the results to be obtained therefrom.

**Die Chirurgische Asepsis der Hände.** Von Dr. MED. WILH. POTEN. Pp. 47. Berlin: S. Karger. London: Williams and Norgate. 1897.—The methods of purifying the hands of the surgeon and gynæcologist have given rise to much discussion of late years, and it is possible that even yet more perfect and simple plans may be devised, but already the results are of immense practical value. The writer gives an interesting account of various plans which have been adopted, and especially discusses the value of disinfection by strong spirit, which he finds very trustworthy. He has now employed it solely for two years, during which he has performed a great number of laparotomies and vaginal hysterectomies. This booklet is well worth the attention of the operator who desires to obtain perfect results from his labour.

**Practical Manual of Diseases of Women and Uterine Therapeutics.** By H. MACNAUGHTON-JONES, M.D. Seventh Edition. Pp. xxiv., 909. London: Baillière, Tindall and Cox. 1897.—From cover to cover this book is essentially practical, but we think it is better adapted for the practitioner than the student. The work has rapidly run through its various editions, and is now half as large again as the fifth edition which was published in 1891. All the recent literature on the subject has been incorporated where necessary to bring it up to date, but the author in emphatic and somewhat racy language clearly expresses his own methods and views. The light vein which runs through the book helps to convey the practical lessons it is intended to teach, as for example where massage was employed in the case of a fibroid tumour, and the author tersely remarks that "the kinetic energy here might have been more safely expended on the lady's boots." The work is profusely illustrated, and will be found full of useful suggestions and sound treatment. We are glad to notice that the list on the title page of the societies to which the author belongs and his appointments now occupies only five lines instead of twelve.

**Some Aspects of Infantile Syphilis.** By J. A. COUTTS, M.B. Pp. 130. London: Rivington, Percival & Co. 1897.—It has become the fashion lately to reprint lectures in book form, and Dr. Coutts's Hunterian lectures of 1896 are worthy of this more lasting memorial. The lectures deal with inherited syphilis and the absolute and invariable truth of Colles's law. Many interesting facts are brought forward to show that the period during which a syphilitic parent may become the father of an infected child is much longer than is usually stated, the author agreeing with the opinions of foreign syphilographers rather than those of the British school headed by Mr. Hutchinson. Dr. Coutts shows that exceptions do exist to Colles's law, but are so few that he does not consider that the practice of allowing healthy mothers to suckle tainted infants should be condemned. The lectures are well worth a careful study, the arguments both for and against being put very fairly and fully, while the author expresses his own opinions frankly in spite of their being opposed to many well-known authorities.

**Aetiologische Studien über Lepra.** Von Dr. EDWARD EHLERS. Pp. 64. Berlin: S. Karger. London: Williams and Norgate. 1896.—In this excellent *brochure* on the subject of the etiology of leprosy, Dr. Ehlers begins by a sketch of our knowledge of the spread of leprosy in the past, and of the resulting distribution of the disease at the present time. He then gives an account of a thorough investigation of leprosy in Iceland derived from personal observations. He found 119 cases: in 56 there was a history of leprosy in other members of the family; in 63 there was none, and contagion was probable in 20 of these, leaving only 43 cases in which no history of contagion was obtainable. Further, he shows that there are innumerable chances of infection both from the want of segregation and from the personal habits of the people. The author comes to the conclusion that leprosy is an infectious disease, and that inheritance plays no part whatever in its production. The booklet is illustrated by twenty-two most vivid representations of the clinical features of the disease, and which even by themselves would render this reprinted article well worth having.

**Résultats de l' Examen histologique de 64 Végétations adénoïdes.** Pp. 30. Bordeaux: G. Gounouilhou. 1896.—**Empyème du Sinus maxillaire chez les Enfants.** Pp. 13. **Pathogénie et traitement des Déviations et Éperons de la Cloison du Nez chez les jeunes Enfants.** Pp. 5. Bordeaux: G. Gounouilhou. 1896. **Traitement de l' Ozène.** Pp. 63. Bordeaux: Feret et Fils. 1897. **De l' Ouverture large de la Caisse et de ses Annexes.** Pp. 65. Bordeaux: Feret et Fils. 1897.—The clinical and operative work of Dr. Moure, the author of these pamphlets, is evidently of a very thorough character. He speaks of fifty-nine open operations on the tympanum and mastoid antrum without a single death as the direct result of the operation. He fully describes the various methods of operating in this region, such as those of Stacke, Schwartze, Kuster, Bergmann, and others, and illustrates his descriptions with notes of cases operated on by himself. His observations are of great interest and value, and we recommend his work to the notice of all surgeons interested in this class of cases. Dr. Moure's remarks on ozæna, or fœtid atrophic rhinitis, indicate that he has spent much time and trouble in investigating this most unsavoury subject. He does not originate any new theory of its etiology, nor does he recommend any novel method of treatment, but he passes in review the voluminous literature of the subject and makes some eminently practical observations. The chapters on adenoids and on empyema of the maxillary sinus in children are well worthy of perusal.

**Transactions of the American Ophthalmological Society, 1896.** Vol. VII., Part III. Hartford: Published by the Society. 1897.—Besides the memorial notice of the late Dr. H. W. Williams, this volume contains forty-three papers, most of them being records of cases. Dr. Bull's paper, comprising a record of thirty-six cases of orbital tumour which have been treated by him, is full of interest, and his conclusions are, in the main, in accordance with, and confirm, the generally accepted views with regard to the risk of interfering with these growths; though we should hesitate to accept the doctrine that the removal of the orbital periosteum hastens the return of the growth by depriving the underlying bone of its protecting envelope. The pathology of some of the tumours is not definitely stated; but, excluding six which appear to have begun as epithelioma of the lids (and therefore subject, surely, to rather different rules of surgical procedure), it is interesting to note that the average duration of life after the first operation was about fifteen months. All the thirty-six cases ended fatally, from coma in only four instances, from exhaustion in nearly all the others. In addition to this series, eight other cases, including one of carcinoma of the choroid, are related in detail by different authors. Dr. J. A. Andrews gives notes and illustrations of an example of tubercle of the iris, and furnishes a brief synopsis of thirty other recorded cases. "Double Chorio-Retinitis following Lightning Flash" is the title of a communication by Dr. C. A. Oliver. There is a want of precision in the notes, but the subjective sensations of the patient are minutely described and depicted. Such details are interesting from a psychological point of view, but should be accepted with the greatest reservation; for, in the first place, the patient was "a twenty-year-old engraver and artist" who previously had complained of muscular and accommodative asthenopia, and was suffering from "a low grade of compound hyperopic astigmatism," the extent of which is not specified. Secondly, there is nothing to show that the changes in the fundus did not exist before the eye was subjected to the flash of lightning, and the statement that "the left eye seemed normal in every respect" is contradicted a little further on by the note, "the nerve-head was a trifle too gray for age, and was somewhat hazy." The line of treatment—confinement to bed with the eyes bandaged, so that they might have thorough rest, and be subjected to unremitting daily examinations—is equally contradictory. There are three papers on glaucoma: in one, Dr.

Richey lays down the proposition that chronic glaucoma is due to some unhealthy digestive process which causes a hyperplasia of the connective tissue of the globe; but he does not adduce any evidence in favour of the proposition. Instances are related in which taxis and manipulation of the globe proved beneficial. There are several other papers of great interest and admirably illustrated, and yet others which are hardly worthy of permanent record, which occupy too much space in a volume which claims to contain the cream of American ophthalmic work.

**Transactions of the Royal Academy of Medicine in Ireland.** Vol. XIV. Dublin: Fannin and Co., Ltd. 1896.—The whole tone of this volume is eminently practical. An interesting case of purulent pericarditis is described by Dr. J. O'Carroll. The pericardium was tapped on the twenty-ninth day of illness, thirty ounces of pus being withdrawn. It re-accumulated rapidly, and on the thirty-fifth day incision was resorted to, the centre of incision being at the seat of puncture, which was in the fourth left space, midway between the sternal edge and nipple. The patient, however, died five weeks later. The pericardium was almost entirely obliterated, and contained only a drachm of pus. Thirteen cases are referred to, of which eleven were treated by tapping, two by incision. Of the whole thirteen only two recovered, being the ones treated by incisions. This mode of treatment is strongly advocated, and seems from the standpoint of theory and practice to be correct. Dr. E. H. Tweedy gives a paper of much interest on the treatment of eclampsia. He condemns chloroform, the induction of labour and especially pilocarpin, and advocates the use of purgatives, morphia, and in some cases venesection. It is a subject on which statistics are difficult to obtain, and not of much value for many reasons; and therefore his paper is not very convincing, although good results by the methods he advocates are brought forward. The volume contains many articles of considerable interest and value.

**Transactions of the Kentucky State Medical Society.** New Series, Vol. V. Louisville: John P. Morton & Company. 1897. —Many interesting topics are treated in this volume in an instructive manner. We would call the attention of surgeons to Dr. L. S. McMurtry's contribution on stricture of the œsophagus and the different methods of performing gastrostomy so as to prevent leakage from the stomach after the operation; and of physicians to a series of papers on various forms of renal disease. The appendix receives the usual amount of attention, and the question of operative or non-operative treatment is discussed with much vigour, and in terms rather astonishing to English readers.

**Reports from the Laboratory of the Royal College of Physicians, Edinburgh.** Vol. V. Edinburgh: William F. Clay. 1894.—Many valuable papers on anatomy, physiology, pharmacology, and pathology will be found in this volume. It is difficult to single out the work of any particular men, but perhaps that of Gillespie on gastric digestion, and that of Boyd on the proteids of the urine in Bright's disease, are of especial interest to ordinary practitioners. Boyd shows conclusively that the transudation of proteids through the kidney in Bright's disease cannot be a simple process of filtration of these from the blood through a damaged epithelium; it is rather one of depraved or diseased secretory activity. Gillespie, amongst other interesting researches, has one on the question whether Ringer's dictum that acids inhibit acid secretions, whilst alkalies increase an acid secretion, and conversely, is true of the gastric as well as of the salivary glands. He finds that alkalies do increase and acids decrease the gastric acidity if given before food. He points out that, since the converse results will occur in the case of the pancreas, a dose of alkali before meals will increase both the gastric and pancreatic secretions, whilst acids act in the reverse direction. Hence, for ordinary intestinal flatulence acids should be given before meals, alkalies after. The volume abounds in good work, which, by increasing our knowledge of normal and pathological processes, gives us a better grip of clinical phenomena and improves our power both of treatment and prognosis.

**The Year-Book of Treatment for 1898.** London: Cassell and Company, Limited.—This now well-established, popular and well-arranged review of the past year's therapeutical progress maintains its well-earned reputation. Most of the contributors of the previous year have again held themselves responsible for their respective sections; but we note that Dr. G. A. Gibson is the author of the section on Diseases of the Heart and Circulation, and Dr. H. P. Hawkins writes on Diseases of the Stomach, Intestines and Liver. The text, which is embellished with many useful drawings and diagrams, contains in a very brief and readable form an enormous amount of valuable information for the practitioner.

**The Stethoscope.** *Bristol Medical Students' Journal.* No. I. January, 1898. Bristol: J. W. Arrowsmith.—The medical students of Bristol manifest an abundance of vitality in many directions. The Debating Society, the Dramatic Society, and the Cricket and Football Clubs must make considerable inroads upon the time which is available after the demands of the routine of the medical course have been satisfied, and now this new literary effort will stimulate all to increased activity in a very praiseworthy direction. The medical student is like the man who "the more he gave away the more he had"; he gives away much time and labour for the benefit of the sick, but his stock of knowledge grows thereby, and so we have no question that the time spent on this journal will be well spent, and will in the end be profitable. We wish our spirited contemporary all success, and hope that it will have a long and useful career.

**The Dentist.** No. 1. Vol. I. January, 1898. London: Hampton and Co.—The first number of this new monthly journal is in good taste, and printed in good style on excellent paper. Its articles are on various aspects of dentistry and form interesting reading. Whether there will be a demand for another dental journal remains to be seen; but there is always room for good things, and we wish the new comer success.

**The Edinburgh Medical Journal.** New Series, Vol. II. Edinburgh: Young J. Pentland. 1897.—We are pleased to congratulate the editor on the appearance of his second volume, which is well printed, well bound, and full of interesting matter. The original papers are numerous and original. The reviews of British and foreign literature are carefully done and signed by the respective writers, and the recent advances in the different branches of medical science are carefully recorded by well-known specialists in the various departments. Dr. Gibson and his staff of coadjutors may be counted upon for much useful work in the future.

**Archives Provinciales de Chirurgie.** Paris: Institut International de Bibliographie Scientifique.—Many of our readers will be glad to know that this well-written and well-illustrated periodical has been added to our exchange-list. It contains much that is original and useful, and little that we need hesitate to copy and advise.

**Burdett's Official Nursing Directory.** Compiled and Edited by Sir HENRY BURDETT. 1898. London: The Scientific Press, Limited.—Modelled somewhat on the same lines as the *Hospitals and Charities*, this work is likely to prove of use. In the making of these directories Sir Henry delights; he has the qualities for it, and probably a well-trained staff. The book contains an outline of the laws affecting nurses, information on the hospitals, on nursing agencies in the mother country and colonies, on provident funds; and also a directory of nurses named alphabetically. There should be also a local list.

Dr. Sajous tells us that *The Annual of the Universal Medical Sciences* is about to begin a modified series, while its companion, *The Universal Medical Journal* (formerly *The Satellite*) is to be continued as *The Monthly Cyclopædia of Practical Medicine*. Instead of giving, as heretofore, only excerpts of the new features of the year, the *Annual* will describe *in extenso* each disease, including its subdivisions: "Etiology," "Pathology," "Treatment," &c. Thus the reader will have before him what in the older work was left to his memory. If the year brings forth anything new it will be inserted in its place in the text. If there be nothing fresh to record upon any particular disease, the account of the latter will, at least, appear as it was when last studied.

Mr. W. B. Saunders, of Philadelphia, sends us a note calling our attention to some of the works he has at press. Amongst them are a volume on Genito-Urinary and Skin Diseases, which is to be issued in the "American Text-Book Series." We have gladly availed ourselves of the opportunity of speaking in the highest terms of many important works in that series, in which several other volumes, we observe with pleasure, are in preparation for early publication. Works on Orthopædic Surgery, Diseases of the Stomach, and Pathological Technique are only some of those that are promised in the year. Mr. Saunders also announces an English Edition of the excellent Atlases published by Lehmann, of Munich, in such admirable fashion and at such moderate prices.

As an evidence of local enterprise we welcome the announcement that Messrs. John Wright and Co. have undertaken the European publication of the *Laryngoscope*, a monthly journal of which upon its appearance in 1896 we spoke approvingly.

